# Selenium-related nutritional status in patients with common variable immunodeficiency: association with oxidative stress and atherosclerosis risk

**DOI:** 10.1186/s12865-021-00425-9

**Published:** 2021-05-13

**Authors:** Itana Gomes Alves Andrade, Fabíola Isabel Suano de Souza, Fernando Luiz Affonso Fonseca, Carolina Sanchez Aranda, Roseli Oselka Saccardo Sarni

**Affiliations:** 1 Department of Pediatrics, Universidade Federal de São Paulo - Escola Paulista de Medicina, Rua Dr. Diogo de Faria, 671, São Paulo, SP CEP 04037002 Brazil; 2 Universidade Federal de São Paulo - Campus Diadema, Diadema, Brazil; 3 Department of Pediatrics, Faculdade de Medicina do ABC/Centro Universitário FMABC, Santo André, Brazil; 4 Department of Pediatrics, Universidade Federal de São Paulo - Escola Paulista de Medicina, São Paulo, Brazil

**Keywords:** Common variable immunodeficiency, Dyslipidemia, Oxidative stress, Cardiovascular risk, Selenium

## Abstract

**Background:**

Common variable immunodeficiency (CVID) is an inborn errors of immunity, that leads to recurrent chronic infections and autoimmune/ inflammatory diseases and neoplasms. It is considered that these condition is related to persistent this immune-inflammatory stimulation and increased oxidative stress. A positive impact on the survival of patients with an inborn error of immunity was observed with advanced clinical care protocols, thus raising concerns about the risk of developing other associated chronic diseases, such as atherosclerosis. Studies suggest that selenium (Se) is a protective trace element against damage caused by oxidative stress. Thus, it is postulated that adequate consumption reduces the risk of some chronic diseases.

**Results:**

Se median levels (ug/L) [45.6 (37.3–56.2) vs. 57.8 (46.0–66.0); *p* = 0.004] and GPX activity (U/L) [7682 (6548–8446) vs. 9284(8440–10,720); *p* = 0,002) were significantly lower in patients compared to controls. Inadequacy of Se levels was observed in 50% of the patients. There was a higher percentage of high values of C-reactive protein in the group of CVID patients compared to controls [8 (36.4%) vs. 2 (11.1%); *p* = 0.082]. Higher concentrations of oxidized LDL (45.3 mg/dL vs. 33.3 mg/dL; *p* = 0.016) and lower concentrations of Apo A-1 (98.5 mg/dL) vs. 117.0 mg/dL; *p* = 0.008) were observed in the CVID group compared to the control. There was a significant and positive correlation between Se plasma levels and apolipoprotein A-1 concentrations in CVID group (rho = 0.577; *p* = 0.001). Se values less than 46 μg / L (OR = 3.590; 95% CI 1.103 to 11.687; *p* = 0.034) and GPX activity below the 4th quartile (OR = 21.703; 95% CI 2.534 to 185.914; *p* = 0.005) were independently associated, after adjustment for age, overweight and dyslipidemia, with the CVID group (Table 5).

**Conclusion:**

This study showed an higher percentage of high us-CRP, lower values of plasma Se and GPX activity, higher concentrations of LDLox and lower levels of Apo A-1 in CVID patients in comparison to controls, suggesting oxidative stress and cardiovascular risk.These data point to the importance of assessing the Se status and cardiovascular risk in these patients.

## Introduction

Selenium (Se) is an essential micronutrient for antioxidant defense that integrates an important part of selenoproteins. The protection provided by Se against dyslipidemia and cardiovascular diseases (CVD) is supported by its role in the antioxidant defense mediated by the glutathione peroxidase (GPX) family. In this context, GPX reduces the formation of hydroperoxides of phospholipids and cholesterol esters, and prevents oxidized low-density lipoprotein (LDL) artery sedimentation and, consequently, slows or prevents the atherosclerotic process [1–3].

The inadequate consumption of Se and the presence of polymorphism in the GPX1 gene are related to the lower activity of this enzyme, which can harm the body’s antioxidant defense system [4]. Some studies have highlighted the relationship between single nucleotide polymorphism (SNP) in GPX genes with increased risk of CVD and metabolic syndrome [5, 6]. The cardioprotective effect of Se is still controversial probably due to the limited trial evidence that is available to date [7].

Common Variable Immunodeficiency (CVID) is a syndrome encompassing a heterogeneous group of diseases characterized by changes in the immune function involving T and B cells, and inadequate production of antibodies, causing recurrent bacterial infections [8]. Because CVID patients may have recurrent or chronic infections and autoimmune/inflammatory diseases and neoplasms, it was suggested that these conditions led to persistent immune stimulation and increased oxidative stress [9]. In a recent study, the analysis of oxidative stress markers in the CVID patients suggested a series of abnormalities in the anti-oxidant system [10].

In this context, Vieira et al. (2015) [11] found increased inflammatory markers and lower high-density lipoprotein cholesterol (HDL-c) and Apoliprotein A-1 (apo A-1) concentrations, suggesting a predisposition to cardiovascular risk in these patients.

A positive impact on the survival of patients with an inborn error of immunity (IEI) was observed with advanced clinical care protocols, thus raising concerns about the risk of developing other associated chronic diseases, such as atherosclerosis.

This study aims to describe the plasma levels of Se and erythrocyte glutathione peroxidase activity in CVID patients and to relate them to lipid status biomarkers.

## Methods

This is a cross-sectional controlled study evaluating 32 patients of both genders aged from 9 to 61 years, diagnosed with common variable immunodeficiency, according to the criteria of the European Immunodeficiency Society, conducted from 2016 to 2019 [12]. The patients were being followed up at the Discipline of Allergy and Clinical Immunology of Federal University of São Paulo, Brazil.

The control group was composed of 37 healthy volunteers matched by age and gender.

Exclusion criteria for patients and controls were considered: presence of acute infection, and use of corticosteroids at the time and in the 3 months preceding the collection of laboratory tests.

The study was approved by the Research Ethics Committee of Federal University of São Paulo (No. 972812/2015), and financed by the São Paulo Research Foundation - FAPESP n° 2015/13308–9. All methods were carried out in accordance with relevant guidelines and regulations including Declaration of Helsinki.

The demographic, clinical, and treatment data were obtained from the patients’ charts. The family history risk of atherosclerosis was assessed for patients and controls.

### Anthropometric and food intake assessment

The anthropometric assessment included measurements of weight, height, and waist circumference. Weight and height were measured according to World Health Organization (WHO) [13].

The body mass index-for-age (BMI/A) and height-for-age (H/A) indicators were calculated for the classification of nutritional status, expressed as Z-score, using De Onis (2007) [14] as a reference, for children and adolescents, and the body mass index (BMI), as proposed by the World Health Organization, for adults (WHO, 1995). The classification by Freedman et al. [15] was used to assess the waist circumference of children and adolescents, and values above the 90th percentile were considered high. Adults were classified according to the WHO [13].

Food intake was assessed with the 24-h dietary recall [16], applied in three times [17], with a 15-day interval. The calculation of energy and nutrients intake was perfomed using the software DietwinR, comparing patients and controls.

Considering that food composition tables available in some software do not have complete data on Se content in foods, these data were included manually based on the article by Ferreira et al. (2002) [18].

### Biochemical assessment

After a 12-h fast, blood was collected to analyze plasma Se, erythrocyte glutathione peroxidase activity, lipid profile, apolipoproteins A-1 and B, oxidized LDL (LDLox), malondialdehyde (MDA), ultra-sensitive C-reactive protein (us-CRP), adiponectin, insulin, glucose, aspartate aminotransferase (AST), alanine transaminase (ALT) and gamma-glutamyl transpeptidase (Gamma GT).

All analyzes were performed using standard methods and good practice in clinical analysis. Plasma Se levels were obtained by atomic absorption spectrophotometry by graphite oven, with a detection limit of 1.0 mcg/L and linearity of 400.0 μg/L. The coefficient of variance was 0.8%. For classification purposes, a cut-off point < 46 μg/L was adopted for inadequacy. Erythrocyte glutathione peroxidase (GPX) activity was measured by the method based on that Paglia and Valentine (RANDOX). The method is linear up to a concentration of 925 U/L. The sensitivity was 75 U/L and the coefficient of variance was 3.4%. GPX activity values lower than 4171 U/L were considered inadequate.

The lipid peroxidation was determined by the TBARS method (thiobarbituric acid-reactive substances) which is based on the reaction of malondialdehyde (MDA), a compound formed by the oxidation of lipids, with thiobarbituric acid (TBA) and is given in MDA equivalents, according to Satoh [19]. Us-CRP was determined using the turbidimetric-immunological method (Roche). The measurement range is 1.0–200 mg/L). Adiponectin levels were measured by ELISA.

The lipid profile, including triglyceride (TG), total cholesterol (TC), and high-density lipoprotein cholesterol (HDL-c) was measured by enzymatic-colorimetric method. LDL-c and VLDL-c were calculated using the formula by Friedewald et al. (1972) [20]. For classification, the cut-off points suggested by the American Academy of Pediatrics [21] and the National Cholesterol Education Program (NCEP) [22] were adopted. The presence of dyslipidemia was considered when the TC > 170 mg/dL for children/adolescents and > 200 mg/dL for adults, or LDL-c > 110 mg/dL for children/adolescents and > 129 mg/dL for adults, or triglycerides > 100 mg/dL for children/adolescents and > 150 mg/dL for adults and/or HDL-c < 35 mg/dL for children/adolescents, < 40 mg/dL for women and < 50 mg/dL for men.

The non-HDL-c cholesterol (NHDL-c) were obtained by subtracting the HDL-c values from the TC values and classified according to the Bogalusa [23] and NCEP. The following atherogenic indices were also calculated: total cholesterol/HDL-c, LDL-c/HDL-c, Apo B/Apo A-1, LDL-c/Apo B, and HDL-c/Apo A-1.

Apo A-1 and Apo B were measured using kits of turbidimetric methods for human Apo A-1 and Apo B (Roche, Indianapolis, IN, USA) and oxidized LDL (LDL ox) by ELISA (Wuhan Fine Biological Technology, Wuhan, China).

Glycemia was measured by enzymatic reference method with hexokinase, while insulin was quantified by electrochemiluminescence. The fasting blood glucose and insulin values were used to calculate the HOMA-IR (Homeostasis Model Assessment of Insulin Resistance) using the following formula: HOMA-IR = fasting glucose (mmol/l) x fasting insulinemia (μU/ml)/22.5.

Alanine transaminase (ALT), aspartate aminotransferase and the gamma-glutamyl transpeptidase were measured by enzymatic colorimetric method.

To evaluate cardiovascular risk we considered inflammatory, oxidative stress and lipid status biomarkers.

### Statistical analysis

The SPSS 25.0 (IBM®) program was used for statistical analysis. Categorical variables were shown as absolute numbers and percentages, compared using the Chi-square test. The continuous variables were evidenced in the median and interquartile range and compared using the Mann-Whitney test. The Spearman test was used to assess the correlation between continuous variables and glutathione peroxidase and Se levels. Logistic binary regression (enter method) was used to evaluate the variables predicting Se levels (< 46 μg / L) and glutathione peroxidase activity (< 1st quartile - <  7384.5 U / L). The model included variables that were clinically relevant - CVID group, overweight, dyslipidemia and age. Significance level adopted in all analyzes was 5%.

## Results

The classification of the nutritional status of CVID patients and the control group is summarized in Table 1. There was no difference between the groups regarding gender, age, pubertal stage, family cardiovascular risk, and BMI. The presence of dyslipidemia was found in 24/32 (75.0%) of the patients.

**Table 1:** Demographic and anthropometric data of CVID patients and the control group

Variables	CVID Patients (*n = 32*)	Control group (*n = 37*)	p
Age, median (IQ_25–75_)	36.8 (27.9–45.2)	34.7 (20.7–44.3)	0.481^b^
Gender			
Male %	14 (43.8)	16 (43.2)	0.972^a^
BMI, classification, kg/m^2^			
Underweight, %	2 (6.2)	1 (2.8)	0.704^a^
Normal, %	15 (46.9)	18 (48.6)	
Overweight, %	15 (46.9)	18 (48.6)	

^a^ Chi-square and ^b^ Mann-Whitney test

### Demographic, anthropometric and clinical data

The characterization of CVID patients is established in Table 2. The median age was 36.8 years (min-max 9.6–61.4 years); there was one child, four teenagers, and the remaining were adults. The delay since onset of symptons and diagnosis was 5 years and 6 months (min-max 0.5–16.4 years). Of the 32 patients, fourteen (43.8%) had a chronic pulmonary disease (CPD) and were on continuous use of antibiotics, while 5/32 (15.6%) had chronic diarrhea. All patients received regular immunoglobulin infusion, and only 7/32 (21.9%) used vitamin or food supplements regularly (data not shown).

**Table 2:** Classification of age, waist circumference and laboratorial variables in CVID patients

Variables (*n = 32)*		N (%)
Age	9–19 years	5 (15.6)
	20–61 years	27 (84.4)
Waist	Adequate	20 (62.5)
circumference	High	12 (37.5)
Lipid profile	High total cholesterol	7 (21.9)
	High LDL-c	6 (18.8)
	High triglycerides	4 (12.5)
	Low HDL-c	18 (56.3)
	High NHDL-c	12 (37.5)
Se levels	Adequate (> 46 μg/L)	16 (50.0)
GPX activity	Adequate (> 4171 U/L)	31 (96.8)

N (%)

*LDL-c* Low-density lipoprotein, *HDL-c* High-density lipoprotein, *NHDL-c* Non-HDL cholesterol, *GPX* Erytrhocyte glutathione peroxidase activity

### Laboratory variables and food intake assessment

Dyslipidemia was observed in the CVID vs. control group, respectively, in 24 (75.0%) vs. 21 (77.8%); *p* = 0.525. Se levels inadequacy was observed in the CVID vs. control group in 16 (50.0%) vs. 6 (22.2%); *p* = 0.036, whereas there was no statistically significant difference between groups for the inadequacy of GPX activity lower values1 (3.1%) vs. 0 (0.0%); *p* = 0.603.

The comparison of laboratory variables is shown in Table 3. Se median levels and GPX activity were significantly lower in patients compared to controls There was a higher percentage of high values of C-reactive protein in the group of CVID patients compared to controls [8 (36.4%) vs. 2 (11.1%); *p* = 0.082].

**Table 3:** Comparison of biochemical variables between CVID patients and control group

Variables	CVID Patients (*n = 32*)	Control group (*n = 37*)	p*
	Median (IQ_25–75_)	Median (IQ_25–75_)	
GPX activity U/L	7.682 (6548–8446)	9.284 (8440–10,720)	**0.002**
Selenium μg/L	45.6 (37.3–56.2)	57.8 (46.0–66.0)	**0.004**
MDA nmol/mL	3.5 (3.1–3.9)	3.2 (2.4–4.0)	0.514
us-CRP mg/L	6.3 (0.9–17.7)	1.8 (0.8–1.8)	0.124
Glucose mg/dL	85.5 (81.0–92.0)	85.0 (77.0–95.0)	0.813
Insulin U/mL	6.6 (2.6–10.8)	8.1 (5.2–13.8)	0.155
HOMA-IR	1.2 (0.5–2.2)	1.6 (0.9–2.9)	0.177
AST U/L	19.0 (15.5–24.0)	18.0 (15.0–19.0)	0.116
ALT U/L	10.0 (8.0–14.0)	12.0 (9.0–17.0)	0.108
GGT U/L	14.0 (10.3–25.8)	16.3 (10.7–21.5)	0.519

Significance level of Mann-Whitney test

*GPX* Erythrocyte glutathione peroxidase, *us-CRP* Ultrasensitive protein C reactive, *MDA* Malondialdehyde, *HOMA-IR* Homeostasis Model Assessment for Insulin Resistance, *AST* Aspartate transaminase, *ALT* Alanine aminotransferase, *GGT* Gamma-glutamyl transferase

In the group of CVID patients, there was no difference between Se concentrations and GPX activity in those with and without chronic lung disease. However, those who had chronic diarrhea had lower Se plasma levels [35.0 (30.4–35.8) vs. 48.0(41.1–61.4); *p* = 0.003] and GPX activity [6379 (6036–6717) vs. 7827 (7086–8756); *p* = 0.030) compared to those who did not have this associated morbidity.

Regarding dietary intake, we observed that the CVID group had a lower intake of polyunsaturated fat (*p* = 0.006) and a higher intake of zinc (*p* = 0.028) and retinol (*p* = 0.026) than the control group. There was no statistically significant difference in Se intake (median) between CVID patients and controls [66.3 (56.7–79.3) vs. 66.2 (60.8–72.3); *p* = 0.682] (data not shown). There was no association between Se intake and age (*p* = 0.197).

The lipid status biomarkers are shown in Table 4. Higher concentrations of oxidized LDL (45.3 mg/dL vs. 33.3 mg/dL; *p* = 0.016) and lower concentrations of Apo A-1 (98.5 mg/dL) vs. 117.0 mg/dL; *p* = 0.008) were observed in the CVID group compared to the control.

**Table 4:** Family cardiovascular risk and lipid status biomarkers of CVID patients and control group

Variables	CVID Patients (*n = 32*)	Control group (*n = 37*)	p*
	Median (IQ_25–75_)	Median (IQ_25–75_)	
Family CVR Yes	13 (40.6)	16 (43.2)	0.826
Lipid status biomarkers			
Total cholesterol mg/dL	166.0 (138.5–185.0)	179.0 (164.0–213.0)	0.085
LDL-c mg/dL	100.1 (86.0–116.6)	108.8 (84.2–139.0)	0.237
Triglycerides mg/dL	91.0 (78.5–104.0)	91.0 (75.0–121.0)	0.995
HDL-c mg/dL	43.0 (34.5–52.5)	48.0 (37.0–56.0)	0.210
NHDL-c mg/dL, %	118.0 (104.0–138.0)	135.0 (108.0–161.0)	0.226
VLDL-c mg/dL	18.2 (15.6–20.8)	18.2 (15.0–24.2)	0.990
Remnant cholesterol mg/dL	18.5 (15.6–20.8)	18.2 (15.0–22.8)	0.909
Oxidized LDL mg/dL	45.3 (26.8–65.7)	33.3 (23.7–42.2)	**0.016**
Apo A-1 mg/dL	98.5 (81.5–112.5)	117.0 (92.0–130.0)	**0.008**
Apo B mg/dL	94.0 (81.0–111.5)	102.0 (90.0–119.0)	0.215
Apo B/ Apo A-1	0.9 (0.7–1.1)	0.8 (0.6–1.0)	0.238
Total cholesterol/HDL-c	4.0 (3.0–4.0)	4.0 (3.0–4.0)	0.484
LDL-c/ Apo B	1.0 (0.9–1.2)	1.0 (0.9–1.2)	0.976
LDL-c/ HDL-c	2.0 (2.0–3.0)	2.0 (2.0–3.0)	0.442
TG/ HDL-c	2.0 (2.0–3.0)	2.0 (1.0–3.0)	0.516
Apo A-1/ HDL-c	2.3 (1.9–2.5)	2.3 (1.2–2.6)	0.392

* Significance level   Mann-Whitney test

*CVR* Cardiovascular risk, *LDL-c* Low-density lipoprotein, *HDL-c* High-density lipoprotein, *NHDL-c* Non-HDL cholesterol, *VLDL-c* Very low-density lipoprotein, *TG* Triglycerides, *Apo A-1* Apolipoprotein A-1

**Fig. 1:**
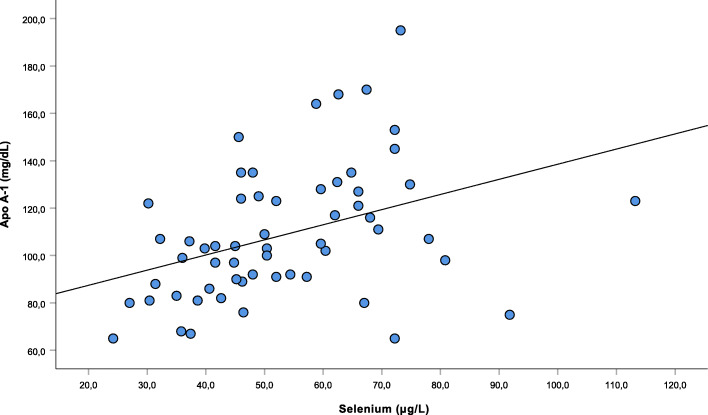
Correlation of Se plasma levels with Apolipoprotein A-1 levels in CVID patients. Level of significance of Spearman’s correlation. Se (ug/L) x Apo A-1 (mg/dL)

There was no significant correlation between GPX activity and the variables studied in CVID patients. In turn, there was a significant and positive correlation between Se plasma levels and apolipoprotein A-1 concentrations in the CVID group (Fig. 1).

Se values less than 46 μg / L (OR = 3.590; 95% CI 1.103 to 11.687; *p* = 0.034) and GPX activity below the 4th quartile (OR = 21.703; 95% CI 2.534 to 185.914; *p* = 0.005) were independently associated, after adjustment for age, overweight and dyslipidemia, with the CVID group (Table 5).

**Table 5:** Predictor variables for selenium and glutathione peroxidase values

Model I		β	Confidence interval 95%		*P* value
Age	years	0.981	0.939	1.026	0.404
Group	CVID	3.590	1.103	11.687	0.034
Dyslipidemia	yes	0.812	0.222	2.968	0.753
Nutritional status	overweight	0.494	0.151	1.613	0.243
Model II		β	Confidence interval 95%		Valor p
Age	years	0.990	0.938	1.044	0.705
Group	CVID	21.703	2.534	185.914	0.005
Dyslipidemia	yes	2.993	0.501	17.881	0.229
Nutritional status	overweight	0.427	0.103	1.761	0.239

Model I: Dependent variable: Selenium levels < 46 μg/L

Model II: Dependent variable: Glutathione peroxidase activity < 1° Quartile (< 7384.5 U/L)

## Discussion

The study showed significantly lower values of Se plasma levels and GPX activity in CVID patients compared to healthy controls. There was a significant and positive correlation between Se levels and Apo A-1 concentrations, a negative reactive acute-phase protein, and the main protein component of the HDL cholesterol fraction.

According to our knowledge, this study is a pioneer in assessing the association between Se concentrations and GPX activity with lipid status biomarkers in CVID patients. A recent paper evaluating 124 men with human immunodeficiency virus (HIV) infection described serum Se deficiency in 65.9% of individuals. There was a significant and negative correlation between the concentrations of Se and pro-inflammatory cytokines (IL-1beta, IL-6, and TNF-alpha). The authors emphasized that Se deficiency has been associated with increased morbimortality and other adverse outcomes in HIV+ individuals [24].

A study recently published by our group in patients with ataxia-telangiectasia (inborn error of immunity and neurodegeneration) showed inadequate Se values in 40% of patients, with a significant association with oxidative stress biomarkers [25].

Inborn errors of immunity have different clinical characteristics, especially recurrent infections linked to incompetence in regulating different homeostatic processes, with an increased risk of the development of tumors and autoimmune diseases [26, 27]. Furthermore, in predominantly humoral deficiencies, patients are exposed to extracellular bacteria and have severe and recurrent sinopulmonary infections requiring hospitalization and frequent antibiotics use. Bacterial infections are associated with a reduction in serum selenium concentrations, and supplementation studies show favorable results [27].

Many bacteria can also synthesize selenocysteine, suggesting that selenoproteins may play a role in bacterial physiology. Simultaneously, the composition of the host’s microbiota is also regulated by the diet Se. Therefore, pathogenic bacteria, microbiome, and cells of the host’s immune system may be competing for a limited supply of Se, which is of even more significant concern in CVID patients [28–30].

A significant and direct correlation was observed between Se and Apo A-1 concentrations. The literature has shown that Apo A-1 and other HDL-c functionality markers are superior to HDL-c concentrations in predicting risk for cardiovascular disease [31].

Se suppresses the activation of pro-inflammatory pathways by chelating free radicals and blocking the nuclear transcription factor NF-kB activation. Changes in the composition and concentration of lipoproteins that occur in inflammation can alter these particles’ function, making them pro-inflammatory. Thus, besides the quantitative alterations, significant changes are also observed in the composition of these lipoproteins, especially in the case of HDL-c that loses its major Apo A-1 protein component, which is replaced by an acute-phase protein, namely, serum amyloid A, which during inflammation, represents 90% of proteins found in HDL-c. Se deficiency can contribute to the disruption of inflammation, contributing to the generation of dysfunctional HDL-c, that is, a pro-inflammatory particle [32, 33].

A significant transition is expected in the microbiota of CVID patients characterized by reduced intra-individual bacterial diversity due to antibiotic use. Some studies suggest a link between immunodeficiency, systemic immune activation, and altered intestinal microbiota [34]. Thus, we can conclude that recurrent infections, inflammation, and changes in the microbiota contribute to high oxidative stress, insulin resistance, and consequent elevated risk of dyslipidemia [35].

CVID patients compared to healthy controls showed in the present study cardiovascular risk evidenced by: higher percentage of high us-CRP, lower values of plasma Se and GPX activity, higher concentrations of LDLox and lower levels of Apo A-1.

Specific LDLox receptors called LOX-1 and SR-PSOX have been isolated. The expression of LOX-1 is found in endothelial cells, smooth muscle cells, and macrophages, while SR-PSOX is expressed in macrophages. LDLox can be produced due to the increased production of reactive species by the mitochondria during oxidative stress and inflammation [36]. Such findings reinforce the atherosclerotic risk of CVID patients.

Publications assessing CVD risk and its interface with Se are scarce in the literature. In a study of 20 CVID patients and 16 healthy controls, Aukrust et al. (1997) [37] reported high concentrations of malondialdehyde (lipid peroxidation biomarker) and reduced homocysteine in the group of patients, suggesting the existence of oxidative stress. Future studies are required to assess the effect of selenium supplementation on CVD risk in CVID patients.

In conclusion, this study showed an higher percentage of high us-CRP, lower values of plasma selenium and GPX activity, higher concentrations of LDLox and lower levels of Apo A-1 in CVID patients in comparison to controls, suggesting oxidative stress and cardiovascular risk. These data point to the importance of assessing the Se status and cardiovascular risk in these patients.

## Data Availability

The datasets used and/or analysed during the current study are available from the corresponding author on reasonable request.

## Abbreviations

ALT: Alanine transaminase
Apo B: Apolipoprotein B
Apo A1: Apolipoprotein A-1
AST: Aspartate aminotransferase
BMI: Body mass index
CVDs: Cardiovascular diseases
CVID: Common Variable Immunodeficiency
CVR: Cardiovascular risk
FAPESP: São Paulo Research Foundation
GGT: Gamma-glutamyl transpeptidase
GPX: Glutathione peroxidase
H/A: Height for age
HDL-c: High-density lipoprotein cholesterol
HIV: Human immunodeficiency virus
HOMA-IR: Homeostasis Model Assessment of Insulin Resistance
IEI: Inborn error of immunity
LDL-c: Low-density lipoprotein cholesterol
LDLox: Oxidized LDL
MDA: Malondialdehyde
NCEP: National Cholesterol Education Program
NFkB: Nuclear transcription fator B
NHDL-c: Non-HDLc
Se: Selenium
SNP: Single nucleotide polymorphism
TC: Total cholesterol
TG: Triglycerides
us-CRP: Ultra-sensitive C-reactive protein
VLDL-c: Very-low-density lipoprotein cholesterol
WHO: World Health Organization
